# Organizational and systems-level barriers and facilitators to health professionals’ readiness to address domestic and sexualized violence: a qualitative study from Nova Scotia, Canada

**DOI:** 10.3389/frph.2025.1598706

**Published:** 2025-09-29

**Authors:** Enrique Villacis Alvarez, Janine Noorloos, Susan J. Wilson, Robert Green, Shelley Fashan, Christine Pritchett, Crystal John, Alexa R. Yakubovich

**Affiliations:** ^1^ Department of Community Health and Epidemiology, Dalhousie University, Halifax, NS, Canada; ^2^ Nova Scotia Health, Halifax, NS, Canada; ^3^ Health Association of African Canadians, Dartmouth, NS, Canada; ^4^ Women's and Newborn Health, IWK Health, Halifax, NS, Canada

**Keywords:** domestic violence, sexualized violence, violence against women, health services, Nova Scotia, Canada

## Abstract

**Introduction:**

Domestic and sexualized violence, including intimate partner violence, are an increasing public health concern across Canada. Beginning with the province of Nova Scotia, several jurisdictions have now declared this violence to be “an epidemic”, with renewed calls for health systems to be part of prevention efforts. Recent research has shown that while many health professionals are seeing cases of violence in their work, their training, resources, and workplace supports are inadequate. The current paper aimed to qualitatively analyze how discourses around domestic and sexualized violence affect health professionals’ readiness to respond.

**Methods:**

From November 2023 to February 2024, we conducted an online, mixed-methods survey of professionals working in health-related fields in Nova Scotia (*N* = 1,649). We qualitatively analyzed responses from 828 participants who answered at least one open-ended question using reflexive thematic analysis within a feminist poststructuralist framework.

**Results:**

We generated two themes in our analysis. The first theme, “inconsistent approaches to addressing violence”, described how many participants were aware of the impacts of violence on their patients but relied on different discourses for whether (or not) the issue falls within their scope of practice. Participants highlighted key organizational challenges limiting their potential responses to violence (e.g., protocols, training, staffing, time constraints). The second theme, “the limits of downstream health responses amid structural barriers”, highlighted how individual health professionals experienced their positions as too “downstream” to provide significant responses to an issue rooted in structural factors (e.g., housing insecurity) that has only exacerbated since the onset of the COVID-19 pandemic. Many participants reflected on how fragmented systems of support may increase the risk of survivors experiencing violence. Respondents expressed frustration as they recounted limited capacity to meet the needs of survivors without social and structural infrastructures.

**Conclusion:**

Our results provide important insights into current organizational and systems-level barriers and facilitators for responding to domestic and sexualized violence among Canadian health professionals. Government and organizational policy should more clearly define how domestic and sexualized violence is within scope of practice for different health professionals, with appropriate, ongoing training and resourcing. Likewise, structural causes of violence must be recognized, both in terms of identifying and supporting patients and communities at greatest risk and creating opportunities for the health sector to be a part of primary prevention efforts.

## Introduction

Domestic and sexualized violence are the most prevalent forms of violence against women (VAW) and are an increasing public health concern across Canada (1, 2). Domestic, or family violence, includes intimate partner violence (IPV), elder abuse, and child abuse, while sexualized violence more broadly refers to any nonconsensual sexual behaviours or acts committed by anyone, regardless of their relationship to the victim (3, 4). Women and gender diverse people face disproportionately high rates of domestic and sexualized violence, causing significant adverse health consequences, such as mental health problems, injury, chronic disease, and reproductive health risks, leading to greater healthcare visits and costs (1, 3, 5–12). The health system is thus a key avenue for prevention and intervention, especially because healthcare is often the only formal service accessed by survivors of violence (13–15).

In Canada, healthcare is managed and delivered at provincial and territorial levels (1). According to the most recent national data, Nova Scotia was the Canadian province with the highest prevalence of self-reported IPV among women (16). The province has also had an annual rate of femicide higher than the national average, including a recent spate of seven intimate partner homicides from October 2024 until March 2025 (17, 18). The largest mass shooting in Canadian history took place in Nova Scotia in April 2020, following a violent attack by the perpetrator on his wife. The resulting inquiry, the Mass Casualty Commission, demonstrated the perpetrator's longstanding history of VAW and recommended increased collaboration between the health system and other social and community services to better prevent and respond to this violence (19). The Nova Scotia Government declared IPV an epidemic in September 2024, with several other Canadian provinces making or deliberating similar bills (20). As a result, public and political pressure is at an all-time high for a stronger health system response towards domestic and sexualized violence in Nova Scotia and across Canada. While there are some clinical guidelines from the two health authorities in Nova Scotia (Nova Scotia Health and IWK Health) around identifying and responding to different forms of violence, including child abuse, sexualized violence, and IPV, recent events and recommendations have highlighted the need for a stronger health system response (19, 21–23).

Past qualitative research in Canadian jurisdictions has demonstrated key challenges in readiness to respond to domestic and sexualized violence including ambiguity over roles and responsibilities and insufficient organizational protocols, training opportunities, time during patient interactions, and referral pathways (24–30). The existing Canadian research has primarily focused on immediate intra-professional and clinical barriers, with a notable lack of system-level analysis that incorporates the perspectives of health professionals across disciplines and roles, as well as intersections with structural drivers of violence, such as housing insecurity, poverty, and other social inequities. There are limited studies investigating preparedness to respond to violence among health professionals in Nova Scotia, with recent quantitative research demonstrating significant gaps (31). In particular, nearly half of the participants in a 2024 study of professionals working in health services and policy across the province reported seeing at least one case of abuse in their work in the past six months. Yet a minority of participants reported that addressing violence was part of their team's goals and most knew nothing or little about their role in responding to or preventing domestic or sexualized violence. The current paper uses qualitative data from this study to explore how health professionals' responses to domestic and sexualized violence are shaped by social and institutional discourses and identify the ways in which these contextual factors can be better accounted for in efforts to strengthen health system responses to violence, in Nova Scotia and beyond.

## Methods

We used data from The Interprovincial Violence Against Women Project (or The IPV Project), a mixed-methods study aiming to strengthen systems responses to VAW across Canada (31). Our research is informed by a transformative worldview, which centres the promotion of social justice through meaningful collaboration, especially with marginalized communities, and advocacy (32, 33). The IPV Project uses an integrated knowledge translation approach with knowledge users from the health and VAW sectors included as partners and collaborators in all stages of the research. Full methodological details have been previously reported (31). Briefly, an approximately 15 min mixed-methods survey was conducted online using REDCap from November 2023 to February 2024 in Nova Scotia. Health professionals working in government, public health, community health, or healthcare organizations over the age of majority (19 years or older) were eligible to participate. The recruitment strategy was multifaceted to reach a large and diverse sample of participants. Study information was shared with eligible participants via health organizations and departments, professional associations and networks, and public registries of licensed health professionals. Specializations more likely to encounter survivors of domestic and sexualized violence were prioritized in recruitment, including, for instance, mental health and addictions, sexual and reproductive health, emergency, trauma, primary healthcare, and public health (14, 34). Estimation of response rates by program areas is available elsewhere (31). The survey instrument was adapted from the Physician Readiness to Manage Intimate Partner Violence Scale (PREMIS) (35) for relevance to both domestic and sexualized violence, multidisciplinary professionals, and the Nova Scotia context. Participants provided informed consent prior to starting the survey and, after completion, could opt into a draw to win one of three $100 gift cards. The Nova Scotia Health Research Ethics Board approved this study (REB#1028425).

The survey had 16 open-ended questions, most of which were only shown to participants depending on survey skip logic (e.g., if a particular response to a close-ended question was selected). After an initial review of all questions, we focused our analysis on five that contained the richest data related to the current research objectives. These questions focused on: the relationship between violence and team goals, patients' health needs, and challenges and opportunities in responding to domestic or sexualized violence (see Table 1 for a summary of the questions and skip logic involved). Participants must have answered at least one of these open-ended questions to be included in the sample.

**Table 1:** Summary of the five open-ended questions under study and the associated survey skip logic.

Close-ended question	Answer options	Follow-up open-ended question
Is addressing domestic or sexualized violence part of your team's objectives or goals?	Yes	**Please briefly describe in what ways this violence is part of your team's objectives or goals.**
	No	**Why do you think domestic or sexualized violence is not part of your team's objectives or goals?**
How many new cases (picked up an acute case, uncovered ongoing abuse, or had a patient/client disclose a past history) of domestic or sexualized violence would you estimate you have seen in your work in the last 6 months?	None	
	1–5	**What have you observed as the greatest health needs among domestic or sexualized violence survivors? If relevant, please describe in what ways these needs have changed since March 2020.**
	6–10	
	11–2-	
	21 or more	
	Don’t Know	
Do you or does your team provide patients/clients who have experienced domestic or sexualized violence with education or resource materials?	Yes, almost always	
	Yes, when it is safe	
	Yes, but only upon request	
	No, due to inadequate referral resources in the community	
	No, because I do not feel these materials are useful in general	
	No, other reason	**Please Specify**
What have been the greatest challenges in collecting this information? Please select all that apply.	Limited funding, resources, or staff time	
	Limited staff training on how to collect or record this information	
	Limited patients/clients capacity to engage in lengthy conversations	
	The method of recording these data is not a good fit with our work (e.g., not mobile)	
	Individuals’ discomfort in providing this information	
	Other	**Please Specify**

The current study used reflexive thematic analysis, which facilitates in-depth engagement with qualitative data to generate organizing concepts (i.e., themes) that summarize the shared meaning in patterns across participants' responses (36–38). This type of thematic analysis emphasises the active role researchers have in creating themes to explain a dataset. We conducted our analysis within a feminist poststructuralist (FPS) framework, which attends to the ways in which language is used in historically situated social and institutional contexts to construct meaning and establish social positions (or subjectivities) and relations of power (39–42). These language practices constitute “discourses” and power is understood as relational, rather than a fixed property ascribed to certain individuals, continually influenced by the social and institutional discourses at hand. FPS has its roots in feminist scholarship focused on understanding and dismantling practices that perpetuate systems of oppression towards women (43–45), but has been widely used in health research with applications to social inequities and power relations beyond gender alone (39, 42, 46–48). Through a FPS framework, we focused our thematic analysis on identifying patterns in participants' reports that highlighted how their responses to domestic and sexualized violence were shaped by social and institutional discourses (36, 39).

Participant responses were extracted from REDCap into a Microsoft Excel spreadsheet. Analysis began with data familiarization, wherein four research assistants manually coded the data (adding codes to adjacent cells) and collaboratively discussed initial observations with the broader research team. Two researchers (EVA and JN) then conducted open semantic and latent coding across the dataset, following a similar process that built upon and developed these initial observations. Each coder added their codes in a separate column of the spreadsheet, which allowed them to collaboratively build on each other's coding as the process evolved. Both researchers wrote down notes and memos in a separate document throughout coding to document their process and developing perspectives. The coding process adhered to the tenets of reflexive thematic analysis, in which coding is not conceptualized as “objective”, but an iterative process where researchers' subjective perspectives are embraced for the interpretation of the dataset (49). Therefore, any differences among the coders were discussed during meetings with the aim of incorporating diverse perspectives into a cohesive thematic framework. After initial open coding, all codes were transferred into a separate spreadsheet, where subsequent code iterations were conducted in different columns to document their evolution. Similar codes were then collated to facilitate the identification of patterns of shared meaning in the dataset. The researchers developed initial themes by summarizing these patterns. From this, a thematic framework was drafted and iteratively and collaboratively refined through ongoing discussions with the broader research team. Each coder revisited the spreadsheet data to conduct focused latent coding to discern participants' values, beliefs, and practices around responding to domestic and sexualized violence, in reference to the study's quantitative findings (31) and existing literature. A FPS lens enabled us to critically explore how health professionals' opinions and experiences of responding to domestic and sexualized violence were socially constructed and the ways in which “the status quo” can be challenged. Coders met regularly with the broader research team throughout the analysis process to refine and finalize the thematic framework.

In this study, participants had different levels of engagement with patients. Some participants worked in direct support roles conducting only patient-facing work, others managed direct support staff, other participants were both patient-facing and managed a team, and finally some participants did neither (e.g., policy development). We considered these distinctions during the data analysis process by exploring potential differences in perspectives within and between these different groups. The thematic framework was shared and finalized with feedback from knowledge user partners, who held varying roles (including healthcare leaders, physicians, clinical therapists, and registered nurses) across different disciplines (e.g., emergency medicine, community health, maternity and childcare). We present participant quotes with identification numbers and contextual information (work role, geographic location, and engagement with patients) throughout the results to support and contextualize our analysis.

## Results

There were 1,649 participants in the survey, 50% of whom (*n* = 828) responded to at least one open-ended question. Table 2 summarizes the sociodemographic and professional characteristics of the 828 participants who responded to at least one open-ended question, compared to the total sample. There were no meaningful differences between these two groups.

**Table 2:** Summary characteristics of the sample.

Characteristic	Current study *N* = 828	Full survey sample *N* = 1,649
Sociodemographics		
Race and ethnicity^a^	(*n* = 728)	(*n* = 1,346)
Only white selected	600 (82%)	1,092 (81%)
Acadian	41 (6%)	85 (5%)
Indigenous	21 (3%)	47 (3%)
Black	12 (1%)	30 (2%)
East or Southeast Asian	9 (1%)	25 (2%)
South Asian	11 (1%)	23 (2%)
Middle Eastern	7 (1%)	19 (1%)
Latino	5 (1%)	15 (1%)
Jewish	5 (1%)	8 (1%)
Other	5 (1%)	8 (<1%)
Prefer not to answer or don’t know	15 (2%)	38 (2%)
Gender	(*n* = 720)	(*n* = 1,333)
Ciswoman	645 (90%)	1,206 (90%)
Gender minority^b^	16 (3%)	25 (2%)
Cisman	59 (8%)	102 (8%)
Sexual identity	(*n* = 723)	(*n* = 1,337)
Heterosexual	606 (84%)	1,143 (86%)
Gay or lesbian	20 (3%)	37 (3%)
Bisexual	48 (7%)	70 (5%)
Queer	30 (4%)	45 (3%)
Not sure, questioning, or other	10 (1%)	25 (2%)
Age, median in years (interquartile range) (605), (1,128)	42.5 (34.5–52.5)	41 (33–51)
Born outside of Canada	(*n* = 725)	(*n* = 1,343)
Yes	65 (9%)	109 (8%)
No	660 (91%)	1,234 (92%)
Education above bachelor's (*n* = 726), (*n* = 1,343)	384 (53%)	594 (44%)
Professional information		
Organization^a^		
Nova Scotia Health	541 (65%)	1,077 (65%)
IWK Health	157 (19%)	293 (18%)
Community health organization or private practice	211 (25%)	392 (24%)
Government	48 (6%)	68 (4%)
Education^a^		
Non-clinical	112 (14%)	329 (20%)
Business administration	18 (2%)	62 (4%)
Health administration	10 (1%)	57 (3%)
Public health	23 (3%)	46 (3%)
Community health	11 (1%)	26 (2%)
Biomedical science	15 (1%)	15 (1%)
Epidemiology	1 (<1%)	7 (<1%)
Law	1 (<1%)	6 (<1%)
Engineering	0 (0%)	5 (<1%)
Other	63 (8%)	145 (9%)
Clinical	716 (86%)	1,320 (80%)
Nurses	326 (39%)	585 (35%)
Physicians (including residents)	114 (14%)	179 (11%)
Social workers	118 (14%)	156 (9%)
Psychologists	50 (6%)	65 (4%)
Healthcare assistants	18 (2%)	69 (4%)
Occupational therapists	18 (2%)	48 (3%)
Counselors	33 (4%)	44 (3%)
Nutritionists/dieticians	18 (2%)	43 (3%)
Physiotherapists	12 (1%)	24 (1%)
Clinical therapists	23 (3%)	26 (2%)
Dentists	6 (1%)	14 (1%)
Midwives	6 (1%)	6 (<1%)
Other	54 (7%)	165 (10%)
Job role^a^		
Direct support to patients, clients, or community members	737 (89%)	1,413 (86%)
Managing or leading people providing direct support	296 (36%)	532 (32%)
No direct support (e.g., policy, research)	45 (5%)	147 (9%)
Specialization^a^		
Leadership	312 (38%)	567 (34%)
Mental health and addictions	228 (28%)	341 (21%)
Primary healthcare	117 (14%)	260 (16%)
Maternity and early years	125 (15%)	193 (12%)
Emergency, critical care, and trauma	109 (13%)	185 (11%)
Long-term and continuing care	61 (7%)	156 (9%)
Public health	73 (9%)	154 (9%)
Community health organization	66 (8%)	103 (6%)
Children's health	47 (6%)	101 (6%)
Surgical services	35 (4%)	83 (5%)
Rehabilitative and restorative care	24 (3%)	60 (4%)
Sexual assault nurse examiner	41 (5%)	52 (3%)
Diet and nutrition	10 (1%)	29 (2%)
Sexual health	16 (2%)	20 (1%)
Senior leadership	10 (1%)	19 (1%)
Safety	5 (1%)	10 (<1%)
Health zone		
Central	321 (39%)	619 (38%)
Western	179 (22%)	334 (20%)
Eastern	141 (17%)	300 (18%)
Northern	143 (17%)	262 (16%)
Provincial	145 (18%)	301 (18%)
Areas serviced or focused on in work		
Only urban	201 (24%)	540 (33%)
Only rural or remote	282 (34%)	378 (23%)
Urban and rural/remote	134 (16%)	255 (15%)
Provincial	190 (23%)	399 (24%)
Not applicable	21 (3%)	77 (5%)
Start date in current role		
Before the COVID-19 pandemic (11 March 2020)	481 (58%)	908 (55%)
After the COVID-19 pandemic	347 (42%)	741 (45%)

^a^ Categories are not mutually exclusive and therefore percentages may not add up to 100.

^b^ Cell sizes are too small (*n* < 5) to parse into more detailed categories.

Values are N (%) unless otherwise noted.

We developed two themes related to social and institutional discourses and participants' attitudes, practices, subjectivity, and agency when responding to domestic or sexualized violence: (1) inconsistent approaches to addressing violence, and (2) the limits of downstream health responses amid structural barriers.

### Inconsistent approaches to addressing violence

Participants' responses to whether addressing violence is part of their team's goals tended to draw upon different discourses centred around why violence is (or is not) within the scope of practice of different health programs or professional roles (see Table 3 for exemplary quotes by extent of patient engagement). At one end of the spectrum, participants discussed addressing violence as intrinsic to healthcare provision and programmatic goals. Participants used words such as *inherent, duty, crucial, and implicit* across their responses to describe how intertwined addressing violence is with their definition of care to patients, clients, or communities. These participants tended to draw upon a more holistic understanding of health, including physical, mental, and social wellbeing. Frameworks aligned with understanding health holistically (e.g., social determinants of health or trauma-informed care) informed how participants described the impact of experiences not traditionally considered in biomedical discourse (e.g., violence) on patients' health. In contrast, many participants who described violence as not being part of their team's goals employed institutional discourses that set out strict scopes of practice—often through a more biomedical approach—that constructed violence as tangential to health problems or clinical work. Many participants appeared to conform to this discourse, in part justifying it with large workloads, greater priorities, and lack of preparation to address the issue.

**Table 3:** Participant quotes demonstrating contrasting perspectives regarding whether violence is or is not part of their team's goals.

Participants’ level of patient engagement	Addressing violence part of participants’ team's goals	Addressing violence not part of participants’ teams’ goals
Only Patient-Facing	“Working in urgent care centers, we work with all different populations experiencing a number of things and being able to identify victims of violence and act accordingly is crucial to our role.” (ID 1428, Registered Nurse in Emergency and Critical Care, Rural or Remote) “Implicit in the work of child and youth mental health is protection of children and youth who may be impacted by any of these forms of violence.” (ID 2101, Mental Health and Addictions Clinician, Rural or Remote)	“Acute care setting therefore not the focus however if it is identified it is addressed to the extent possible” (ID 1744, Health Care Social Worker in Primary Care, Urban and Rural areas) “We provide family-based care, however given the patient is [a pediatric] patient, so unless the violence is a part of the formulation for the child's needs, it does not become a treatment priority nor are staff provided with adequate training to manage the potential of domestic violence should it arise.” (ID 1052, Social Worker in Mental Health and Addictions, Urban and Rural areas) “It would be if it [violence] presented itself otherwise it is not the focus of our service.” (ID 1648, Clinical Social Worker in Primary Care, Provincial)
Patient-Facing & Manages Team	“Part of the awareness and assessment of children at all parts of care. Pediatricians are trained in identifying and responding to concerns re child abuse and hence, domestic violence.” (ID 584, Pediatrician, Provincial) “Ensuring staff are prepared to care for patients with pre-existing trauma is part of our orientation programs. Trauma informed approaches are used in all policy and education development and implementation.” (ID 850, Clinical Educator in Perinatal Care, Urban)	“Our current scope is already very large” (ID 639, Director/Senior Leadership in Sexual Health, Urban) “Focus is on provision of mental health services” (ID 2053, Psychiatrist in Community Mental Health, Urban) “I wouldn’t say it's not relevant to the type of work we do, but not identified as an objective per se. I think we informally screen for this and are aware but could probably do a better job of addressing this.” (ID 1053, Physician in Cancer Care and Perinatal Care, Provincial)
Only Manages a Team	“It is part of our vision and mission to promote health and wellness of all within the scope of the Soc. Determents [sic] of health” (ID 660, Director/Senior Leadership in Community Health, Urban) “Our team focuses on care of ppl who have experienced a recent sexual assault but as part of that, it is often combined with DV [Domestic Violence], IPV [Intimate Partner Violence], and HT [Human Trafficking]. We deal with the mental and physical after effects [sic], treat within our scope, and refer ppl [sic] to other organizations that can help.” (ID 210, Manager/Nurse in Sexual Assault Nurse Examiner Program, Provincial)	“Other NSH [Nova Scotia Health] department responsibility/accountability” (ID 161, Manager/Team Lead in Primary Care, Rural/Remote) “We have a very focused mission to prevent unintentional injury” (ID 18, Leader in Population Health Initiatives, Provincial)
Neither Patient-Facing nor Managing a Team	“Trauma informed lens to clinical guidelines and resources. Incorporate trauma informed care approach to education and include content specific to developmental trauma, gender and sexualized violence, and domestic violence.” (ID 63, Nurse Consultant in Perinatal Care, Provincial) “Addressing freedom from violence and discrimination as a significant determinant of mental health” (ID 15, Health Promotion Specialist in Mental Health and Addictions, Provincial)	“Not a key priority right now” (ID 429, Program Officer in Public Health, Provincial) “Goals are higher level, not issue specific” (ID 595, Senior Leadership in Public Health, Rural or Remote) “Team prioritizes clinical/medical issues” (ID 846, Nurse Consultant in Perinatal Care, Provincial)

Participants working within and across health programs exhibited these differences in perspectives. Some participants worked in programs designated to address specific forms of violence, and thus easily saw the relevance of violence to their work, such as participant 210, a sexual assault nurse examiner (see Table 3). However, other participants who worked in similar areas of the health system held contrasting views around whether responding to violence is relevant to their scope of practice. For instance, participants 1428 and 1744 both worked in acute care settings in patient-facing roles. Yet the former described why violence is considered a goal by identifying and acting on violence as “crucial” to their role, while the latter emphasized that, because they worked in an acute care setting, violence was not a direct responsibility to clinical care. These contrasting responses were found across participants in similar health programs, demonstrating the individual, rather than systematic, ways health professionals conceptualized their role in responding to violence.

The existence of protocols, guidelines, mandates, or clarity of programs' goals was a key factor guiding the discourses that informed participants' responses to violence. For instance, participant 903 referred to their organizational policy when describing the ways that violence is part of their team's goals:

“Our organization has a policy to immediately report any suspected elder abuse to management, and we have compulsory seminars on abuse in the workplace” (ID 903, Personal Support Worker, Rural or Remote, Only Patient-Facing)

Participants who embraced violence among their goals and had more structured protocols on responding to violence often provided more elaborate responses as to how violence should be considered in their workplace contexts and what kind of responses they can provide. For instance, participant 385 shared their views on how IPV should be considered a goal within emergency departments:

“The Emergency Department is the entry point to the healthcare system. Staff care for many marginalized and at-risk populations. It is important for them to recognize the signs of IPV to ensure that patients are provided with appropriate supports, that duty to report requirements are met for vulnerable populations, and that their physical assessment is reflective of screening for the risks of IPV and considers potential physical and emotional implications of IPV”. (ID 385, Nurse, Urban, Patient-Facing and Manages a Team)

Participant 385 and many others (e.g., 1428), demonstrated a social construction of violence as a priority in emergency departments via a “high-risk” discourse, wherein violence was understood as more likely to affect populations experiencing other forms of marginalization. Participant 385 highlighted how responding to violence included connecting people to appropriate support so that they could receive holistic care beyond their immediate needs. In a separate response, the participant indicated their knowledge and connection with these supports: “*Reporting to Child Welfare/Protection Consulting SANE for patients who have experienced sexual assault*”. The awareness of their location, relational interactions with groups at risk of violence, and knowledge of available resources informed how this participant prioritized addressing violence in their practice. Others drew upon a discourse of strict scopes of practice when they recounted the chaotic nature of the emergency department as an entry point to the health system. Many of these participants reflected on this context as a place that leads to overwhelming feelings and a de-prioritization of “non-medical” issues.

As mentioned above, understandings of violence and responses were not uniformly shared among those in similar contexts, such as participant 1532, who was a coordinator of clinical operations also working in an emergency department. When asked why violence was not considered a priority among their team's goals, they responded: “*Done as individuals. NO screening done at triage. MUST reinforce importance of documentation with ER nurses constantly. It does not appear to a priority to protect at risk patients*”. Participant 1532's response signals a social discourse wherein violence is devalued across emergency departments while demonstrating their resistance to this discourse, through constantly reinforcing the importance of violence identification and management to their colleagues. In their reflection, the participant highlighted individuality in staff's responses to violence rather than standardized procedures. Participant 1532 used capitalized words to share their frustration at the lack of a systematic response to protect those at higher risk of violence since they placed high value on responding to violence within the emergency care context. For these participants, the lack of organizational directives or their implementation suggested that violence was not valued or prioritized throughout their programs. Participants in other health programs similarly highlighted dominant discourses influencing responses to violence across the health system, wherein violence is not prioritized as a health issue of focus, leaving (in)action to individuals' discretion. Participant 411 reflected on the lack of a systematic response to violence by emphasizing how individual support is prioritized:

“Not an area of focus for the programs we currently offer; supported individually but not a targeted issue through current programs within our team”. (ID 411, Program Manager, Rural or Remote, Patient-Facing and Manages a Team)

Beyond immediate screening and connection to resources, participants who considered violence a priority among their teams described more proactive practices towards violence. Through proactive strategies, providers were able to exert their agency by creating safer spaces for patients where they could discuss potential experiences of violence. For instance, participant 606, a clinical therapist, reflected how they would “*allow a safe space for individuals to disclose and or report*” when thinking about how violence was a part of their team's goals. Some participants highlighted how employing more proactive violence-informed strategies could facilitate safety and comfort in their patients with more opportunities for disclosures of violence. Participant 1782 shared the importance of these proactive strategies as: “*Clients need to feel comfortable with the provider they are with to speak freely*” (License Practical Nurse, Urban and Rural areas, Only Patient-Facing). While participants like 606 and 1782 valued their strategies to facilitate proactive discussions on violence, others placed the responsibility of initiating these discussions on their patients. For instance, participant 1648 explained the ways that violence would become a focus of their service:

“It would be if it [violence] presented itself otherwise it is not the focus of our service”. (ID 1648, Clinical Social Worker, Provincial, Only Patient-Facing)

Here, the participant calls upon a discourse wherein clinically relevant violence is thought to *present itself*, which would presumably involve a patient speaking out for themselves or having visible signs of violence. This pattern disregards competing discourses that prevent or delay patients' disclosures of violence (e.g., including normative conceptions of “victims” that invoke shame, fear of child apprehension). For participants like 1648, addressing domestic and sexualized violence among their patients would take priority only once those signs are present, leading to potential missed opportunities for prevention and support. This variability across participants' practices reinforces the pattern of an inconsistent approach within and across programmatic areas, which puts the onus of a system response on individual professionals. Many participants were left to develop individualized responses to violence without appropriate systematic processes for support. For instance, participant 303 explained how their capacity to respond to domestic and sexualize violence is limited as they: “*Don’t have enough time or resources to address this on top of other clinic's focus. Always extremely short staffed*” (ID 303, License Practical Nurse, Rural or Remote, Only Patient-Facing). Like participant 303, there were references embedded across participant responses of an under-resourced health system limiting participants' capacity to incorporate violence-informed approaches in their practices. These experiences were predominantly shared when explaining why violence is not part of their team's goals or the kind of resources or educational materials participants have available to share in their practice. For instance, participant 1706 described the organizational barriers limiting their capacity to respond to violence:

“We’re aware of these issues and don’t really have the best training on how to navigate them when they come up” (ID 1,706, Registered Nurse, Urban, Patient-Facing & Manages a Team)

Participants like 1706 shared a systematic lack of training opportunities, staffing issues, time, and workplace support to feel knowledgeable and prepared around domestic and sexualized violence. Participants who discussed challenges with resources to address violence shared frustration in their limited capacity for responding to violence as they recognized the importance of adequate resources. Health professionals were left in a position where they already did not have adequate resources to address patients' immediate health needs, much less an issue that many considered outside their scope of practice. These experiences pointed to an institutional discourse in which participants cited the lack of appropriate resources to consider violence within a lengthy task list. Despite awareness of the potential health consequences of violence in some cases, many participants lacked an environment in which risk factors and signs could even be explored in patient interactions or programmatic focus.

### The limits of downstream health responses amid structural barriers

This theme explores how many participants, particularly those in direct patient-facing roles, experienced the limitations of a downstream health system in addressing the structural conditions underpinning violence. While managers and leaders often acknowledged the importance of addressing domestic and sexualized violence through their programs, patient-facing professionals' narratives drew upon a discourse of constrained capacity and lack of systemic supports when navigating their patients' complex realities. In this context, “downstream” refers to determinants of health and wellbeing that are closer to the individual, such as clinical services, behavioural interventions, or immediate crisis responses, and that are shaped by upstream social, economic, and political forces. The healthcare system is generally conceptualized as an intermediary determinant of health (50), meaning it operates downstream from broader structural factors like poverty, housing policy, or systemic violence. At the same time, the health system also includes health policy and governance structures, which are more accurately situated as upstream.

Many participants described tensions between the dominant discourse of the health system situated too downstream with their upstream conception of the health system. Participants navigated power relations in which they were positioned too downstream to address the complex needs of patients experiencing violence. In addition to considering complex psychosocial needs of patients, participants like 806 highlighted the need for increased intersectoral supports:

“Health needs are affected by the clients ability to feel safe and this requires consideration to housing, transport, basic needs like food etc., but also the removal of the abuser, access to appropriate and ongoing (not short term) support services and multi agency response to address and support victims and their children it is not just one agencies role to address these issues”. (ID 806, Clinical Therapist & Social Worker, Rural or Remote, Only Patient-Facing).

These participants emphasized that patients' health needs were heavily influenced by unmet basic necessities, such as housing, food, transportation, and safety, and that meaningful care often required coordinated, multi-agency responses. Through their responses, participants negotiated power relations as they sought to expand the dominant discourse of a biomedical approach to patient care by prioritizing other needs (e.g., psychosocial) beyond immediate health ones. Many participants highlighted how health needs are exacerbated by the few supports available to address basic necessities which, at times, were perceived by patients as inadequate or even harmful. For instance, participant 1400 recounted how their capacity to making changes in patients' situations was restricted:

“Quite often the social situation of the patient is such that making a change is very difficult and the option of a shelter is seen as worse than what they are suffering already”. (ID 1400, Emergency Physician, Urban, Only Patient-Facing).

Participant 1400 and others underscored how complex patient needs and the lack of appropriate supports limit the effectiveness of healthcare interventions and leave providers feeling frustrated. As one trauma therapist explained: “*The greatest health needs are basic needs such as housing, food and financial as insecurity in these areas create barriers to doing therapeutic work*”. (ID 1855, Trauma Therapist, Rural or Remote, Only Patient-Facing). These participants were frustrated, given how many of the challenges their patients face stem from long-standing, pervasive social inequities. They also recounted limited options to support patients experiencing violence, knowing that once the clinical interaction ends, those individuals often remain highly vulnerable due to the absence of structural support. This frustration was particularly pronounced among participants in patient-facing roles, who felt they had little control over the system-level supports that could meaningfully improve their patients' lives through upstream interventions.

Participants frequently identified housing as one of the most urgent yet unmet health needs for survivors of violence. Safe and stable housing was described as a precondition for safety planning and recovery, especially when individuals were at risk of re-entering dangerous environments. One registered nurse emphasized the broader implications of housing precarity when describing the greatest health needs of survivors of violence, explaining: “*To have safe housing if partners are being de-incarcerated—the survivors’ safety is now jeopardized and put in situations they were not expecting. Without enough housing, we are doing a disservice to our society, by knowingly re-traumatizing people and putting their health at risk*”. (ID 16, Registered Nurse, Urban and Rural areas, Only Patient-Facing). Without addressing structural determinants of violence, many participants felt that survivors of violence will remain at increased risk of violence and poor health outcomes. Even when housing or financial abuse were identified as issues during clinical interactions, many participants reported a lack of appropriate resources to support their patients. As one participant explained: “*Sometimes housing and financial abuse comes up and we have little to no resources to help*”. (ID 169, Psychologist, Provincial, Patient-Facing & Manages a Team). These gaps in care pathways left many providers feeling helpless, particularly when they were expected to intervene in complex social issues with limited tools.

Many participants described how the COVID-19 pandemic exacerbated these longstanding structural inequities. Across both urban and rural contexts, providers reported rising rates of food and housing insecurity among patients, which in turn increased their risk of experiencing violence. A psychologist reflected on how pandemic-related stressors have increased vulnerability to domestic and sexualized violence: “*From my perspective, the number of patients and families struggling with food and housing insecurity has increased significantly since the COVID-19 pandemic. These added vulnerabilities affect individuals living with domestic or sexualized violence to a greater extent for a number of reasons (e.g., harder to move out and find housing, shelters are full, families are under greater stress leading to more abuse, etc.)*” (ID 1733, Psychologist, Provincial, Only Patient-Facing). Others echoed similar concerns, noting the intensified pressure on families has left survivors of violence with increased experiences of violence and reduced services for support.

Intersecting forms of marginalization, particularly substance use and mental health challenges, further complicated access to support services. Several participants working in inpatient psychiatry or emergency care described how patients were routinely discharged into unsafe environments because shelters would not accept individuals who were actively using substances. These gaps in care pathways were described as harmful, given how they could place patients experiencing domestic or sexualized violence in more violent situations. One participant described the consequences of these gaps in care:

“A lot of patients are not safe at home and there are no safe houses or shelters for them to go to. A large component of this is that a large majority of patients use substances and no shelter or safe house that we do have will take anyone under the influence of a substance. This is a big barrier to patient safety. We need to create safe houses/shelters that allow people using substances to stay at. There are not enough detox beds in this province to simply send everyone to detox first”. (ID 302, Nurse, Rural or Remote, Only Patient-Facing).

Like participant 302, many others identified access to mental health services as critical, yet often inaccessible, when discussing the greatest health needs among patients experiencing domestic and sexualized violence. They described long wait times, the absence of trauma-informed providers, and rigid models of care that failed to accommodate the realities of ongoing violence and complex trauma. Respondents such as 1113 and 1528 stressed the importance of developing services that reduce isolation and strengthen community connections to address these gaps in mental health care, particularly those that are flexible, culturally responsive, and low barrier:

“MH [mental health] support, reducing isolation, making connections with community—accessibility to be able to do that.” (ID 1,113, Community Home Visitor, Rural or Remote, Only Patient-Facing);

“Access to low barrier, trauma informed, flexible therapy” (ID 1528, Mental Health Clinician, Urban, Only Patient-Facing).

These responses emphasized a shared concern: without timely, trauma-informed mental health care, survivors of violence remain unsupported in their recovery and vulnerable to continued harm. The cumulative lack of social and structural support, such as housing, income security, transportation, and mental health, undermined the health system's ability to meet the needs of survivors of domestic and sexualized violence. Participants' frustration over the lack of options for their patients highlighted how providers felt restricted to only short-term immediate responses during their patient interactions. This theme illustrates how the downstream positioning of frontline care, when disconnected from upstream policy and intersectoral coordination, limits both providers' capacity to respond to violence and patient outcomes.

## Discussion

Our results provide critical insight into the reasons health professionals may or may not incorporate addressing domestic or sexualized violence into their practices and opportunities for restructuring a stronger health system response to violence. Our findings demonstrated that participants who reported readily addressing violence in their practices were commonly influenced by a more holistic construction of health [e.g., calling upon frameworks of 'social determinants of health' or “trauma-informed” approaches (50, 51)]. In contrast, participants often relied on an institutional discourse dictating strict “clinical” or “medical” scopes of practice to justify why violence falls outside of their professional goals, in line with previous qualitative research that has shown how many health professionals conceptualize violence as outside of their responsibilities or competencies (24, 25, 52–54). In the current study, we found that many participants across this spectrum of personal practices highlighted organizational and systems-level challenges to violence response that were identified in recent quantitative research (31), including a lack of clear organizational guidelines, competing priorities with scarce resources, or limited training on how to support survivors of violence. However, among those participants who viewed health holistically, these barriers often served as a point of tension to practicing in ways that they viewed as upholding their professional duties; whereas for those relying on strict scopes of practice, these barriers were often cited as justifications for the need to focus on what was seen as patients' “immediate” health needs.

Our findings further demonstrate that even within similar health departments in Nova Scotia, responses to violence, including screening and referrals, are not homogenous, resulting in individualized responses rather than a systematic approach. These individualized responses signal potential missed opportunities to systematically support survivors of violence, with some providers describing proactive approaches to building trust and facilitating disclosures of violence while others carried expectations that violence would “present itself” as relevant. Implementing official guidelines along with adequate and routine training on responding to violence could improve the capacity of health professionals in patient-facing positions to identify and discuss violence in non-judgmental and compassionate ways, which may support survivors in feeling more encouraged to share their experiences (14, 25, 54–60). Likewise, formalized policies by health leaders and managers could convey an institutional commitment to addressing these forms of violence by ensuring a functional health infrastructure that facilitates a systematic response across programs (14, 58, 60–62). Mobilizing a society-wide response that includes the health system requires a greater investment of organizational resources to foster an environment where health professionals know their roles and feel empowered to respond to domestic or sexualized violence.

Our results also provide insights into the limited capacity to respond to violence that health professionals experience in the context of health and social crises. Many participants expressed frustration over the limited response they could provide to problems rooted in socio-structural and historical disparities. Overall, participants in more frontline positions viewed themselves as locked within a subject position too “downstream” to make significant impacts in a complex issue like violence. In particular, our analysis highlighted a layered sense of constraint among health professionals, who described a disconnect between the available “downstream” responses to violence and the broader structural conditions that shape patients' safety, health, and recovery. In the absence of upstream action, coordinated systems, and policy-level change, participants reported feeling ill-equipped to respond meaningfully to the structural “upstream” drivers perpetuating violence. Experiences around limitations on care provision and the associated negative emotions (e.g., frustration) have been described as moral distress (i.e., being unable to act on identified problems due to challenges outside of one's control) (63, 64). A recent systematic review and meta-analysis found a significant positive correlation between moral distress and emotional exhaustion (63) which is a key indicator of burnout among health professionals (65). Increased experiences of emotional exhaustion are problematic for individual providers (e.g., fatigue, psychological exhaustion), and also for the health system as they are linked with intention to leave a position or the profession, job dissatisfaction, and quality of patient care (63, 64).

Participants highlighted the intertwined relationship between health and psychosocial disparities survivors of violence experience, such as mental health conditions, chronic illnesses, and housing and food insecurity (3, 5, 7, 14). Respondents remarked on a fragmented care pathway that places survivors at increased risk of experiencing violence, particularly for referrals to housing and mental health. It is necessary for the health system to coordinate with other social systems to provide a more comprehensive immediate and sustained response to survivors and increase the capacity of health professionals (66). This type of intersectoral work needs clear collaboration guidelines, funding availability, and alignment of agendas to support survivors properly (67). Despite the need for increased intersectoral collaboration, participants in our study expressed disappointment over the limited social support infrastructure to care for the needs of survivors, which have only been exacerbated since the onset of the COVID-19 pandemic. Standardized safety pathways for survivors require integrated government, health, and social infrastructures that address not only immediate needs, but also the structural inequities that sustain violence (3, 68–70).

Our study has some limitations. Findings from this investigation may not be transferable beyond the sample. Participants may be more committed to the issue of domestic and sexualized violence; thus, our sample may have higher knowledge and motivation to address violence than other health professionals in the province. This limitation may indicate that the challenges reported in our study could be even more pronounced outside of our sample. In addition, survey responses tend to provide less contextual information (e.g., shorter responses; no non-verbal cues) compared to other forms of qualitative data collection. However, the current survey was able to reach a large proportion of participants across Nova Scotia's health system (the largest Canadian sample to date on health professionals' readiness to respond to violence). Lastly, around half of the survey participants did not complete any open-ended question; nonetheless, those that did were remarkably similar to the overall sample in terms of their sociodemographic and professional characteristics.

## Conclusion

Findings from our study highlight key organizational and systems-level barriers and facilitators affecting the responses to domestic and sexualized violence among health professionals in Nova Scotia, Canada. Health professionals' qualitative experiences suggest a need for clearer governmental and organizational policies regarding scopes of practice as these relate to violence, along with ongoing training and organizational resources. Similarly, socio-structural factors of violence must be accounted for to identify and support those at increased risk of violence, as well as to expand the health sector's role in prevention efforts. Strengthening health system responses to domestic and sexualized violence, despite a recognized need, will require both an intersectoral approach and clear organizational directives that delineate responsibility across identification and referral pathways, with appropriate system-level supports.

## Data Availability

The datasets presented in this article are not readily available because Individual qualitative responses are not available to ensure participant anonymity. Requests to access the datasets should be directed to alexa.yakubovich@dal.ca.
